# Detection of extended-spectrum β-lactamase-producing *Escherichia coli* genes isolated from cat rectal swabs at Surabaya Veterinary Hospital, Indonesia

**DOI:** 10.14202/vetworld.2023.1917-1925

**Published:** 2023-09-21

**Authors:** M. Thoriq Ihza Farizqi, Mustofa Helmi Effendi, R. Tatang Santanu Adikara, Ira Sari Yudaniayanti, Giovanni Dwi Syahni Putra, Aswin Rafif Khairullah, Shendy Canadya Kurniawan, Otto Sahat Martua Silaen, Safira Ramadhani, Saumi Kirey Millannia, Sergius Erikson Kaben, Yusac Kristanto Khoda Waruwu

**Affiliations:** 1 Department of Veterinary Medicine, Faculty of Veterinary Medicine, Universitas Airlangga. Jl. Dr. Ir. H. Soekarno, Kampus C Mulyorejo, Surabaya, 60115, East Java, Indonesia; 2 Division of Veterinary Public Health, Faculty of Veterinary Medicine, Universitas Airlangga. Jl. Dr. Ir. H. Soekarno, Kampus C Mulyorejo, Surabaya, 60115, East Java, Indonesia; 3 Division of Veterinary Anatomy, Faculty of Veterinary Medicine, Universitas Airlangga. Jl. Dr. Ir. H. Soekarno, Kampus C Mulyorejo, Surabaya, 60115, East Java, Indonesia; 4 Division of Veterinary Clinic, Faculty of Veterinary Medicine, Universitas Airlangga. Jl. Dr. Ir. H. Soekarno, Kampus C Mulyorejo, Surabay, 60115, East Java, Indonesia; 5 Division of Animal Husbandry, Faculty of Veterinary Medicine, Universitas Airlangga. Jl. Dr. Ir. H. Soekarno, Kampus C Mulyorejo, Surabaya, 60115, East Java, Indonesia; 6 Department of Animal Sciences, Specialisation in Molecule, Cell and Organ Functioning, Wageningen University and Research. Wageningen, 6708 PB, Netherlands; 7 Department of Biomedical Science, Faculty of Medicine, Universitas Indonesia, Jl. Salemba Raya No. 6 Senen, Jakarta, 10430, Indonesia

**Keywords:** cat, *Escherichia coli*, extended-spectrum β-lactamase, multidrug resistance, public health

## Abstract

**Background and Aim:**

*Escherichia coli* causes a bacterial illness that frequently affects cats. Diseases caused by *E. coli* are treated using antibiotics. Because of their proximity to humans, cats possess an extremely high risk of contracting antibiotic resistance genes when their owners touch cat feces containing *E. coli* that harbor resistance genes. This study was conducted to identify multidrug-resistant *E. coli* and extended-spectrum β-lactamase (ESBL)-producing genes from cat rectal swabs collected at Surabaya City Veterinary Hospital to determine antibiotic sensitivity.

**Materials and Methods:**

Samples of cat rectal swabs were cultured in Brilliant Green Bile Lactose Broth medium and then streaked on eosin methylene blue agar medium for bacterial isolation, whereas Gram-staining and IMViC tests were conducted to confirm the identification results. The Kirby–Bauer diffusion test was used to determine antibiotic sensitivity, and the double-disk synergy test was used to determine ESBL-producing bacteria. Molecular detection of the genes *TEM* and *CTX-M* was performed using a polymerase chain reaction.

**Results:**

Based on morphological culture, Gram-staining, and biochemical testing, the results of sample inspection showed that of the 100 cat rectal swab samples isolated, 71 (71%) were positive for *E. coli*. Furthermore, 23 *E. coli* isolates (32.39%) demonstrated the highest resistance to ampicillin. Four isolates were confirmed to be multidurg-resistant and ESBL-producing strains. Molecular examination revealed that three *E. coli* isolates harbored *TEM* and *CTX-M*.

**Conclusion:**

In conclusion, pet owners must be educated on the use of antibiotics to improve their knowledge about the risks of antibiotic resistance.

## Introduction

Cats are domesticated animals that frequently interact directly with humans [1]. More than 50,000 kittens are born daily, increasing the cat population [2]. However, only 20% of cats are kept in homes, and the remaining 80% are feral; moreover, 6–8 million cats enter animal shelters annually [3]. To maintain pets healthy and clean and prevent disease, it is essential to take good care of them [4]. Bacterial infections might cause cats to become sick [5]. *Escherichia coli* is a bacterium that frequently affects cats [6], which is because most *E. coli* bacteria are typical flora that are found in the digestive tracts of both humans and animals, but some of them are pathogenic and can cause diarrhea [7]. Although antibiotics are used to treat illnesses caused by *E. coli* [8], their use should not be arbitrary because unnecessary and continued use can result in new issues [9]. Antibiotic resistance is demonstrated by the ineffectiveness of antibiotics used to treat patients with bacterial infections [10]. Based on the experience of treating patients in several animal hospitals, a number of patients showed no improvement after receiving antibiotic treatment [11]. Animals and the environment are believed to be one of the reservoirs for developing resistant bacteria that can either directly or indirectly infect people [12].

*Escherichia coli* can be resistant to specific antibiotics even when it has never been in contact with them because other bacteria that have previously been resistant could have passed the resistance gene to it [13]. Because of their proximity to humans, cats possess an extremely high risk of contracting antibiotic resistance genes when their owners touch cat feces containing *E. coli* that harbor resistant genes [14]. Pet owners risk becoming sick if they do not wash their hands after touching the cat and accidentally swallow microorganisms from their hands [15]. Subsequently, it may be challenging to treat a sick person when a bacterial ailment develops [16]. Multidrug resistance (MDR) occurs due to the inappropriate use of antibiotics that causes resistance in bacteria [17]. Rzewuska *et al*. [18] discovered that 66.8% of *E. coli* isolates were multidrug-resistant to antibiotics from the classes of penicillin, cephalosporin, fluoroquinolones, aminoglycosides, and tetracycline (TE). Hence, MDR will exert a detrimental effect on the choice of therapies to be applied to manage the illness.

Resistance to third-generation cephalosporins generally occurs in Gram-negative bacteria due to extended-spectrum β-lactamase (ESBL) enzyme production [19]. Extended-spectrum β-lactamase-producing bacteria are found in humans, pets, and the environment [20]. Two major ESBL-coding genes, TEM and CTX-M [21], produce ESBLs that hydrolyze β-lactam antibiotics [22]. Extended-spectrum β-lactamase-producing bacteria generally exhibit a phenotype resistant to multiple antibiotics [23]. This situation poses a challenge for infection management in clinical practice. Surabaya, a major city in Indonesia, boasts several animal hospitals, including the Animal Husbandry Service Animal Hospital of East Java Province and the Airlangga University Educational Animal Hospital. In the animal hospitals of Surabaya, studies on *E. coli* resistance are still infrequently conducted. As cats are particularly susceptible to *E. coli* infections of the digestive system, it negatively affect the choice of medicine to be used for disease control [24]. A significant problem affecting the health of people, animals, and the environment is antibiotic resistance that develops in veterinary facilities [25].

This study aimed to identify multidrug-resistant *E. coli* strains and ESBL-producing genes from cat rectal swabs collected at Surabaya City Veterinary Hospital to determine antibiotic sensitivity, which would be useful for reducing the development of antibiotic resistance and improving the effectiveness of treatment.

## Materials and Methods

### Ethical approval

Cat rectal swab samples were used in this study; hence, ethical approval was not necessary. Cat rectal swab samples were collected from Airlangga University Teaching Animal Hospital and Animal Husbandry Service Animal Hospital of East Java Province, Indonesia, during routine case handling.

### Study period and location

This study was conducted from March to April 2023. Cat rectal swab samples were collected at the Airlangga University Teaching Animal Hospital and the Animal Husbandry Service Animal Hospital of East Java Province, whereas bacterial isolation and sensitivity tests were conducted at the Veterinary Public Health Laboratory, Faculty of Veterinary Medicine.

### Sample collection

Approximately 100 samples of cat rectal swabs were collected. Swab results were labeled, and swab sampling was performed carefully. A tube containing Brilliant Green Bile Lactose Broth (BGBLB) (Oxoid™ CM0031, UK) was placed over the cotton swab holding the sample. The samples were transported to the laboratory and placed in a cooler box.

### Isolation and identification

Each cat rectal swab sample was cultured in 10 mL of BGBLB enrichment media and incubated for 18–24 h at 37°C. Positive results are characterized by a change in media color from green to cloudy green and the presence of gas in the Durham tube [26]. Next, 100 μL of the cultured sample was streaked onto eosin methylene blue agar (EMBA) (Oxoid™ CM0069) and incubated for 18–24 h at 37°C. Purification and IMViC tests were conducted to identify three to five *E. coli* colonies that were metallic green in color [27]. In addition, Gram staining was performed for microscopic observation.

### Antibiotic sensitivity test

The Kirby–Bauer diffusion test was conducted to determine antibiotic sensitivity. *Escherichia coli* isolates were planted on agar plates using a sterile cotton swab with a standard McFarland turbidity of 0.5 and evenly rubbed on Mueller Hinton Agar (MHA) (Oxoid™ CM0337B) media surface [28]. The MHA medium had antibiotic disks implanted on it, which were subsequently incubated for 24 h at 37°C. Aztreonam (ATM) 30 μg, ampicillin (AMP) 10 μg, gentamicin (GM) 10 μg, TE 30 μg, and chloramphenicol (C) 30 μg were used for this sensitivity test. The antibiotic disks were placed apart from one another by 25–30 mm. The measurements of the diameter of the inhibitory zone were interpreted according to guidelines based on the Clinical and Laboratory Standard Institute [29].

Multidrug-resistant *E. coli* isolates were subjected to the double-disk synergy test (DDST) using antibiotic disks containing amoxicillin–clavulanate 20/10 μg, cefotaxime 30 μg, and ceftazidime 30 μg. After overnight incubation at 37°C, the diameter of the inhibitory zone was measured to interpret the data.

### Molecular detection

Before testing specific primers for *CTX-M* and *TEM* (Table-1) [2, 30], as reported by Ali *et al*. [30], the first step of DNA extraction from bacterial culture was performed according to Kristianingtyas *et al*. [31], with a few minor modifications to cycling conditions. Deoxyribonucleotide triphosphates, buffers, and Taq DNA polymerase were purchased from Thermo Fisher Scientific Inc., USA, for use in the polymerase chain reaction (PCR) mixture. The thermocycling reaction was performed in 30 cycles of initial denaturation at 94°C for 2 min, followed by annealing at 52°C for 30 s, prolonged at 72°C for 45 s, and a final extension at 72°C for 5 min. Mini-gel electrophoresis was performed to observe the PCR products, and results were recorded using a gel documentation system (Promega, USA).

**Table-1:** Details of primers used in this study.

Primers	Sequences (5’ to 3’)	Target gene	Amplicon size	Reference
*TEM* forward	ATA-AAA-TTC-TTG-AAG-ACG-AAA	bla*_TEM_*	1086 bp	[2]
*TEM* reverse	GAC-AGT-TAC-CAA-TGC-TTA-ATC	bla*_TEM_*		
*CTX-M* forward	CGC-TTT-GCG-ATG-TGC-AG	bla*_CTX-M_*	550 bp	[30]
*CTX-M* reverse	ACC-GCG-ATA-TCG-TTG-GT	bla*_CTX-M_*		

## Results

### Isolation and identification

**Figure-1:**
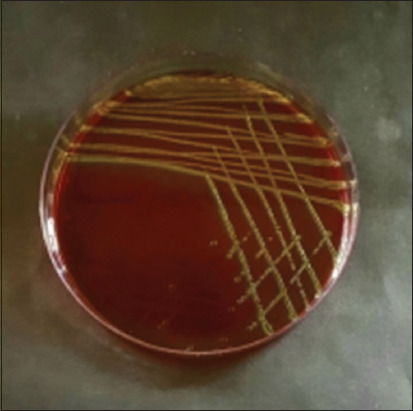
*Escherichia coli* colonies in eosin methylene blue agar.

**Figure-2:**
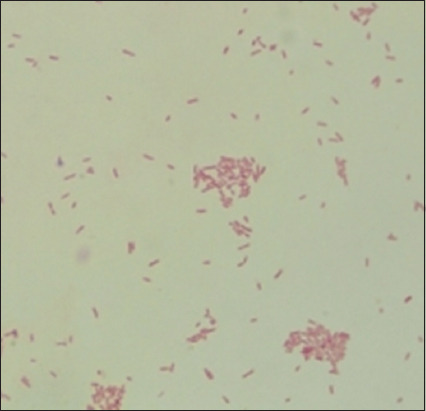
Gram-stained *Escherichia coli* colonies under a microscope.

**Figure-3:**
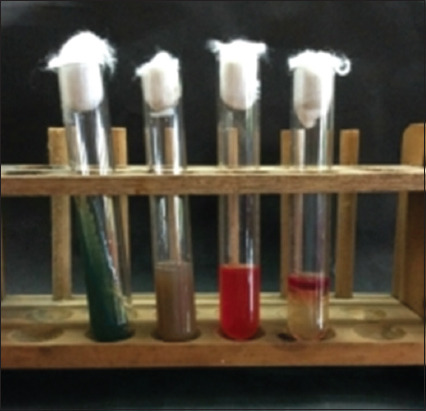
IMViC test results indicate *Escherichia coli* positivity.

Based on morphological culture, Gram-staining, and biochemical testing, the results of sample inspection revealed that of the 100 cat rectal swab samples collected, 71 (71%) were positive for *E. coli* (Table-2). The emergence of metallic green bacterial colonies on the EMBA medium suggested a successful morphological culture of *E. coli* (Figure-1). The presence of red colonies and short rods in Gram-staining indicated a negative Gram-staining result (Figure-2). An indole ring on the SIM test (Indol-positive), an inverted spruce formation on the SIM test (Motil), a red color change on the methyl red (MR) test (positive MR), a yellow color on the Voges–Proskauer (VP) test (negative VP), and green in the citrate test (Citrate negative) were all signs indicating that the IMViC test detected *E. coli* (Figure-3).

**Table-2:** Isolation of *E. coli* from a sample of a cat rectum swabs at a veterinary hospital.

Location	Sample size	Identification test							Positive *E. coli* (%)

		EMBA	Gram- stain	IMViC test					

				Indol	Motile	MR	VP	Citrate	
Airlangga University Educational Animal Hospital	50	45	45	37	37	37	37	37	37 (74)
East Java Provincial Animal Husbandry Hospital	50	47	47	34	34	34	34	34	34 (68)

% (percentage of positive). MR=Methyl red, VP=Voges–Proskauer, EMBA=Eosin methylene blue agar, *E. coli=Escherichia coli*

### Antibiotic resistance

A total of 23 isolates (32.39%) of *E. coli* demonstrated the highest resistance to AMP. This study also determined the level of *E. coli* resistance to numerous additional antibiotics, including five isolates (7.04%) to TE, five isolates (7.04%) to C, four isolates (5.63%) to GM, and three isolates (4.22%) to ATM (Table-3).

**Table-3:** Resistance status of *E. coli* to several antibiotics.

Type of antibiotic	*E. coli* sensitivity status (n = 71)		

	Resistant (%)	Intermediate (%)	Sensitive (%)
ATM	3 (4.22)	1 (1.41)	67 (94.37)
GM	4 (5.63)	2 (2.82)	65 (91.55)
AMP	23 (32.39)	3 (4.22)	45 (63.38)
C	5 (7.04)	3 (4.22)	63 (88.73)
TE	5 (7.04)	7 (9.86)	59 (83.10)

% (percentage of resistant). *E. coli=Escherichia coli*, ATM=Aztreonam, GM=Gentamicin, AMP=Ampicillin, C=Chloramphenicol, TE=Tetracycline

**Figure-4:**
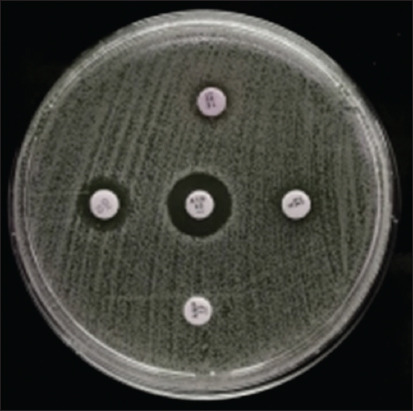
Analyze the susceptibility to antibiotics of an *Escherichia coli* isolate cultured on Mueller Hinton Agar.

The profile of antibiotic resistance based on the results of the *E. coli* resistance test to antibiotics demonstrated that of the 71 *E. coli* isolates, 16 (22.53%) were resistant to one class of antibiotics, whereas 5 isolates (7.04%) were resistant to two classes of antibiotics, and 4 isolates (5.63%) were confirmed to have MDR because they were resistant to three to five classes of antibiotics (Figure-4). The pattern of antibiotic resistance was as follows: ATM–AMP–C, GM–AMP–C, GM–AMP–TE and ATM–GM–AMP–C–TE, each with one isolate (Table-4).

**Table-4:** Isolated *E. coli* resistance profile by antibiotic group.

Group of antibiotics	Resistance profile	Number of isolates (n = 71)	Total number of isolates (%)

		Resistant isolates (%)	
0	No one is resistant	46 (64.79)	46 (64.79)
1	AMP	14 (19.72)	16 (22.53)
	C	1 (1.41)	
	ATM	1 (1.41)	
2	AMP–TE	3 (4.22)	5 (7.04)
	AMP–C	1 (1.41)	
	GM–AMP	1 (1.41)	
≥3	ATM–AMP–C	1 (1.41)	4 (5.63)
	GM–AMP–C	1 (1.41)	
	GM–AMP–TE	1 (1.41)	
	ATM–GM–AMP–C–TE	1 (1.41)	

ATM=Aztreonam, GM=Gentamicin, AMP=Ampicillin, C=Chloramphenicol, TE=Tetracycline, *E. coli=Escherichia coli*

**Figure-5:**
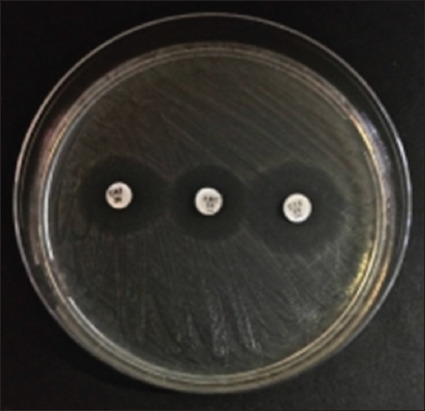
Double-disk synergy test results on extended-spectrum β-lactamase-producing *Escherichia coli* isolates.

Four cat rectal swab samples collected from the Surabaya City Veterinary Hospital contained multidrug-resistant *E. coli* isolates (Table-5). In the DDST on *E. coli* isolates from cat rectal swab samples, four MDR-positive *E. coli* isolates that had been tested (A20, A25, B5, and B6) were positive for ESBL (Figure-5). This could account for the four isolates from the 100 cat rectal swab samples investigated in this study, indicating the low frequency of multidrug-resistant and ESBL-producing *E. coli* infections in Surabaya.

**Table-5:** *Escherichia coli* isolates with a profile MDR and ESBL.

Location	Sample code	Resistance profile	Antibiotic					DDST test

			ATM	GM	AMP	C	TE	
Airlangga University Educational Animal Hospital	A 20	ATM–AMP–C	✓	–	✓	✓	–	Positive
	A 25	GM–AMP–C	–	✓	✓	✓	–	Positive
East Java Provincial Animal Husbandry Hospital	B 5	GM–AMP–TE	–	✓	✓	–	✓	Positive
	B 6	ATM–GM–AMP–C–TE	✓	✓	✓	✓	✓	Positive

✓=Resistant, ATM=Aztreonam, GM=Gentamicin, AMP=Ampicillin, C=Chloramphenicol, TE=Tetracycline, *E. coli=Escherichia coli*, MDR=Multidrug-resistant, ESBL=Extended-spectrum β-lactamase, DDST=Double-disk synergy test

### Molecular detection

**Figure-6:**
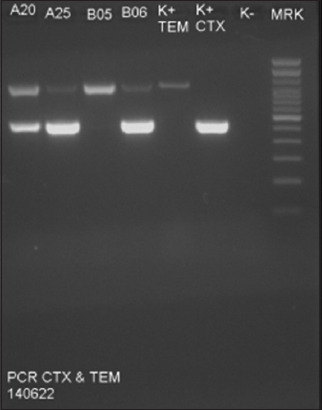
Electrophoresis results of *TEM* and *CTX-M* gene detection. K+=Positive control, K-=Negative control, A20, A25, B05 and B06=Sample code; MRK=Marker.

According to the electrophoresis results, four positive *E. coli* isolates harbored *TEM* and three positive isolates harbored *CTX-M* (Table-6). Moreover, three positive *E. coli* isolates, A20, A25, and B6, harbored both *TEM* and *CTX-M*. The detection of *TEM* and *CTX-M* was indicated by the appearance of a single band in the electrophoresis results (Figure-6).

**Table-6:** Molecular detection of *TEM* and *CTX-M* isolate *E. coli* genes.

Sample code	TEM genes	CTX-M genes
A 20	Positive	Positive
A 25	Positive	Positive
B 5	Positive	Negative
B 6	Positive	Positive

*E. coli*=*Escherichia coli*

## Discussion

### Bacterial isolates

Rectal swab samples collected from cats were used to isolate and identify *E. coli* strains, and 71 of 100 samples collected at the Airlangga University Educational Animal Hospital and the Animal Husbandry Hospital of East Java Province were found to be positive for *E. coli*.

Lactose-containing EMBA medium was used for primary isolation. If the culture contains *Escherichia* *coli*, the acid produced during lactose fermentation will produce green colonies with a metallic shine [32]. When observed under a 1000× magnification microscope, Gram-stained *E. coli* showed a short rod-shaped morphology and were red in color. This is because the thin nature of the cell wall of Gram-negative bacteria, which is composed of lipoprotein and peptidoglycan, prevents *E. coli* from retaining the crystal violet dye during the Gram-staining procedure [33].

The results of indole testing of *E. coli* were positive as proven by the presence of a red ring at the top after adding Kovac’s reagent, and there was turbidity that resembled an upside-down fir tree in the needle-piercing area. The resulting turbidity is evidence of the ability of *E. coli* to move on a semisolid substrate [34]. Tryptophanase, an enzyme produced by *E. coli*, hydrolyzes the amino acid tryptophan into indole and pyruvic acid, causing the formation of the red ring [35].

A positive MR test result is indicated by a hue switch from yellow to red. Because bacteria oxidize glucose by generating acid, the MR test result turns red [36]. A pH MR indicator was added after incubating the media for 48 h. The pH of the media can decrease to ≤4.4 as a consequence of glucose fermentation and production of a variety of acidic chemicals by *E. coli* [37].

After dilution with α-naphthol and 40% KOH solution, the results of the VP test revealed negative findings indicated by a yellow-brown tint. This is because of the ability of *E. coli* to digest carbohydrates into acidic compounds rather than neutral products such as acetonin [38].

The result of the citrate test conducted using Simmons citrate agar (SCA) (CM0155 Oxoid™) media after a 24 h incubation period at 37°C is indicated in green. However, no color change was observed in the citrate test medium, indicating negative results for *E. coli*. This is because *E. coli* bacteria do not utilize citrate as a source of carbon in the environment [39].

### Antibiotic resistance

The Kirby–Bauer diffusion method was used to determine bacterial sensitivity on MHA medium using a standard McFarland 0.5 suspension of the bacterium. The inhibitory zone was measured using a caliper, which was then compared with the 2020 CLSI standard after incubating the media for 24 h at 37°C. Of the 71 isolates, 4 were labeled as multidrug-resistant because they were resistant to at least three to five different classes of antibiotics.

There were 23 isolates (32.39%) exhibited the maximum levels of antibiotic resistance to AMP. The β-lactam class of penicillin derivatives, which includes AMP, prevents the production of mucopeptides required for bacteria to produce their cell walls [40]. These findings are consistent with those reported by Bourne *et al*. [41], who found that 31.1% of *E. coli* isolates from healthy cats had acquired resistance to AMP. This is possible because AMP is a first-line antibiotic, meaning it is the first antibiotic used to treat *E. coli* infections [42]. Moreover, using AMP in the wrong doses or for a prolonged time might lead to significant levels of resistance to the antibiotic [43].

In the antibiotic sensitivity test, the same results were obtained with the antibiotics C and TE, that is, five isolates (7.04%) were resistant. The production of the enzyme C acetyltransferase by *E. coli*, which can decrease antibiotic activity, is the mechanism underlying C resistance [44]. Meanwhile, TE exhibits efflux pump activity, ribosome protection, and enzymatic inactivation according to the primary mechanism of TE resistance [45].

Four *E. coli* isolates (5.63%) demonstrated resistance to GM, an antibiotic of the aminoglycoside class. Zhou *et al*. [46] reported that <10% of *E. coli* isolates exhibited resistance to GM. Kakoullis *et al*. [47] reported that the presence or absence of certain protein receptors on the 30S subunit of 10 ribosomes determines microbial chromosomal resistance to aminoglycosides. The development of adenylated, phosphorylated, or acetylated enzymes, which can break down the medication, is another factor in bacterial resistance to aminoglycosides [48].

In the present study, three isolates (4.22%) exhibited resistance to ATM, which is quite low because of the extremely low usage of the monobactam class of antibiotics in both veterinary and human medicine [25]. The mechanism of ATM resistance involves the destruction of antibiotics by β-lactamases, which causes bacteria to develop antibiotic-resistant cell walls and reduced membrane permeability [49].

The following five different antibiotic preparations were used to test *E. coli* isolates in this study: ATM preparation for the β-lactam monobactam group, GM preparation for the aminoglycoside group, C preparation for the C group, TE preparation for the β-lactam penicillin group, and AMP preparation for the β-lactam penicillin group. Four *E. coli* isolates that were subjected to the antibiotic sensitivity test were found to be multidrug-resistant. Wibisono *et al*. [50] recommended that antibiotics be used at a minimum dosage to attain therapeutic dosages to eliminate and prevent germs from developing antibiotic resistance. Using antibiotics over the recommended dosage might potentially subject bacteria to increased selection pressure, resulting in bacterial mutation and eventual resistance [51].

Regarding the ESBL screening test results for *E. coli*, an ESBL is present when the diameter of the zone of inhibition for drugs containing β-lactamase inhibitors. This is because the β-lactamase inhibitor diffuses into the media and detects the presence of β-lactamase around cefotaxime or ceftazidime [43]. This is indicated by the formation of a zone or is said to be synergistic if a clear zone is found on the edge of the cefotaxime and ceftzidime disk, which widens until the disk contains amoxicillin–clavulanic acid. This synergistic condition is classified as the ability to produce ESBL.

### Molecular detection

The genes *TEM* and *CTX-M* were detected in the rectal swabs of cats in this study. According to a study in China, the epidemiological distribution of *CTX-M* is comparable between animals and humans [52]. Extended-spectrum β-lactamase genes are frequently detected on plasmids that are easily transmissible within or between various bacterial species, and the transfer of genes from humans to animals or vice versa has been the subject of significant reports over time [19]. Reports of genetic resemblances and the discovery of plasmids in humans and animals have been published [22]. According to numerous studies, plasmids transfer horizontally between groups, causing humans in close contact with animals to carry the same strains or strains with the same plasmids [53].

In samples A20, A25, and B6, this study also discovered a combination of *TEM* and *CTX-M*. A similar study by Musa *et al*. [54] found that 8% of participants harbored *CTX-M*, and 77.4% harbored both *TEM* and *CTX-M*. This is because the plasmid that encodes *CTX-M* is a form of large IncFII plasmid that also contains a gene for resistance to other antibiotic classes. Some *CTX-M* genes are associated with IncFII or IncI1 plasmids [52]. The most common type of IncFII plasmid is F2: A-: B-, which has also been found to be associated with *CTX-M bla* in Enterobacteriaceae isolates from other countries [55]. Other isolates that harbor *CTX-M* are highly transmissible, which enables the rapid and effective spread of resistance [56]. Most bacteria that express *CTX-M* are multidrug-resistant. Because plasmids, which are mobile genetic components, include *TEM*-forming genes, they can spread rapidly. Li *et al*. [22] mentioned that *CTX-M* is also associated with *TEM*, *OXA*, and *aac-(6)lbr* on the IncFII plasmid, which carries more than one replicon. A strong promoter exists in the insertion region of *CTX-M*, which is present in several clinical isolates [57].

Antibiotics are administered to animals at veterinary hospitals alone, in combination with other antibiotics, or in conjunction with chemotherapeutics [58]. Extended-spectrum β-lactamase-producing *E. coli* is among the bacteria that are frequently discovered to be resistant to β-lactam antibiotics, aminoglycosides, TEs, and C, which are frequently used in veterinary hospitals [59]. The mechanism underlying resistance in *E. coli* is caused by several processes, including the closure of cell wall pores, which reduces the quantity of antibiotics passing through the cell wall, and an increase in the synthesis of periplasmic β-lactamases, which damages the structure of β-lactams, increasing the pump output (efflux mechanism) on the transmembrane, which allows bacteria to excrete antibiotics before the perception of any effects [60]. Alterations in enzyme activity also prevent antibiotics from interacting with target sites, which inhibits antibiotic action, and lipopolysaccharides prevent antibiotics from binding to targets [61].

In addition to their presence in feces, *E. coli* strains that are resistant to multiple antibiotics can be identified in urine samples, digestive tract, skin wounds, liquid waste, and wounds from surgery [62]. The transfer of genetic elements allows the exchange of genetic material between *E. coli* strains, which facilitates the exchange of antibiotic resistance genes [63]. Bacteria belonging to Enterobacteriaceae can transmit these genes through horizontal transmission between pets and their owners [64].

Both public health and animal health welfare depend on the prudent use of antibiotics to prevent the development of antibiotic resistance [65]. Animal owners who frequently take their pets to the veterinarian do not administer antibiotics appropriately because they believe that medications are no longer required if their pet has healed [66].

## Conclusion

Of 100 cat rectal swab samples collected at Surabaya City Veterinary Hospital, 71 *E. coli* isolates were discovered in the isolation and identification tests. Four multidrug-resistant and ESBL-producing *E. coli* isolates were detected in the sensitivity test and DDST. Based on molecular examination, three *E. coli* isolates harbored *TEM* and *CTX-M*. Therefore, pet owners must be educated on the use of antibiotics to increase their knowledge regarding the risks of antibiotic resistance.

## Authors’ Contributions

MTIF, SR, YKKW, GDSP, and SEK: Collection and/or assembly of data. ARK, SCK OSMS, and SKM: Analysis and interpretation of data and drafted the manuscript. MTIF and MHE: Performed the study. ISY, RTSA, and MHE: Concept anddesign of the study, critical revision of the manuscript. All authors have read, reviewed, and approved the final version of the manuscript.
